# Micronutrient Status in Children Suffering From Anorexia Nervosa: A Cohort of 349 Patients in a Referral Center for Eating Disorders

**DOI:** 10.1002/eat.70120

**Published:** 2026-05-17

**Authors:** Zenaida Iordan, Diane Morfin, Marion Bernetiere, Marie Fauchet, Noël Peretti, Bérénice Segrestin, Pierre Poinsot

**Affiliations:** ^1^ Hospices Civils de Lyon, Centre Référent pour l'Anorexie Mentale et Les Troubles Des Conduites Alimentaires de Lyon Bron France; ^2^ Département Femme Mère Enfant Service de Pédiatrie, Centre Hospitalier Universitaire Vaudois Lausanne Switzerland; ^3^ Hospices Civils de Lyon, Service d'Hépatologie, Gastro‐entérologie et Nutrition Pédiatrique Bron France; ^4^ Laboratoire CarMeN, INSERM‐U1060, INRA‐U1397 Pierre‐Bénite France; ^5^ Centre de Recherche en Nutrition Humaine Rhône‐Alpes, INSERM, INRAE, Université Claude Bernard Lyon 1, Hospices Civils de Lyon Pierre Bénite France; ^6^ Hospices Civils de Lyon, Service d'Endocrinologie, Diabétologie et Nutrition Pierre‐Bénite France

## Abstract

**Objective:**

Anorexia nervosa (AN) is a serious illness in which more than half of all deaths are due to malnutrition. Critically low energy and protein intake are known causes of massive weight loss, whereas micronutrient deficiencies due to a low‐calorie food pattern remain poorly characterized in children with AN. Micronutrient deficiencies in AN, such as selenium deficiency, are known to increase anxiety and the risk of suicide. Two large studies in adults highlighted frequent selenium and copper deficiencies; this information is lacking in children. The present study aimed to provide a comprehensive description of the micronutrient status in pediatric AN and evaluate whether deficiencies differ according to AN subtype (restricting, R, binge‐eating/purging, BP).

**Method:**

A retrospective, single‐center, descriptive study was conducted in a cohort of children with AN who were evaluated at a specialist eating disorders center from 2016 to 2022.

**Results:**

The sample comprised 349 patients (mean age 14.7 ± 1.8 years); 91.4% had AN‐R, 8.6% BP. Mean weight loss was 18.2% ± 10.7%, and initial BMI was 16.7 ± 1.1 kg/m^2^ according to the International Obesity Task Force. At least one micronutrient deficiency was found in 90% of patients; 51.3% experienced multiple deficiencies. The most common were selenium (23.5%), copper (18.4%), and vitamin A (29.1%). The prevalence of deficiencies was similar between AN‐R and AN‐BP, except for potassium and calcium which were significantly lower in AN‐BP.

**Discussion:**

Micronutrients deficiencies are frequent in pediatric AN with no difference according to subtype. The functional impact and the benefit of supplementation remain to be studied.

## Summary

- Children with anorexia nervosa (AN) have a high prevalence of copper and selenium deficiency.
- There is no difference between AN subtypes (restricting, binge‐eating/purging) regarding the prevalence of micronutrient deficiencies.
- Micronutrient status needs to be part of the initial screening in children with AN.

**Abbreviations**
ALAT: Alanine Aminotransferase
AN: Anorexia Nervosa
AN‐BP: Anorexia with Binge Eating and Purging
AN‐R: Anorexia Nervosa with Restrictive Eating
ASAT: Aspartate Aminotransferase
BMC: Bone Mineral Content
BMD: Bone Mineral Density
BMI: Body Mass Index
DBP: Diastolic Blood Pressure
DSM: Diagnostic and Statistical Manual of Mental Disorders
DXA: Dual‐energy X‐ray Absorptiometry
ED: Eating Disorders
FFM: Fat‐Free Mass
FM: Fat Mass
FSH: Follicle‐Stimulating Hormone
FT3: Free Triiodothyronine
FT4: Free Tetraiodothyronine
GFR: Glomerular Filtration Rate
GH: Growth Hormone
HDL: High‐Density Lipoprotein
HR: Heart Rate
IGF‐1: Insulin‐like Growth Factor‐1
IOTF: International Obesity Task Force
LDL: Low‐Density Lipoprotein
LH: Luteinizing Hormone
N: Number
N/A: Not Applicable
PT: Prothrombin Time
SBP: Systolic Blood Pressure
SD: Standard Deviation
T: Body Temperature
TSH: Thyroid‐Stimulating Hormone

## 1 Introduction

Anorexia nervosa (AN) is a serious psychiatric illness associated with high morbidity and mortality (Auger et al. 2021). The Diagnostic and Statistical Manual of Mental Disorders, 5th edition (DSM‐5) identifies two types: a restricting (AN‐R) and a binge eating/purging (AN‐BP) (American Psychiatric Association et al. 2013). Both forms of AN are defined by an inability to maintain an adequate weight for height, due to a critical reduction in energy intake, a fear of gaining weight, and disturbance in body perception. The profound weight loss often is accompanied by severe chronic undernutrition (Alexandre 2019).

The lifetime prevalence of AN is reported to be between 1.2% and 3.3% in females and 0.03%–0.2% in males (Galmiche et al. 2019). Adolescence is a critical period of growth and development, accompanied by a significant increase in caloric and nutritional requirements; malnutrition can lead to deficiencies in iron (causing anemia), vitamins B12 and B9 (causing growth retardation), vitamin C (causing scurvy), and selenium (causing cardiomyopathy) (Berger and Shenkin 2024; World Health Organization 2026). Furthermore, micronutrient deficiencies play a role in thousands of biochemical reactions and have been linked to the pathophysiology of various psychiatric disorders, including AN (Rucklidge et al. 2024). Two studies have shown high rates of micronutrient deficiencies among adults with AN, with at least one deficiency found in 92.8% and 43.7% of patients, depending on the study, with some variations depending on the AN subtype (Achamrah et al. 2017; Hanachi et al. 2019). To date, no medication or micronutrient supplementation has been approved for the treatment of AN, and nutritional rehabilitation remains the priority. However, micronutrients, such as selenium, zinc, and vitamin D, deficiencies have been identified as contributing factors of anxiety and depression, two common comorbidities of AN (Benton and Cook 1991; Hawkes and Hornbostel 1996; Ghaemi et al. 2024; Davarinejad et al. 2026). In addition, selenium deficiency has been associated with an increased risk of suicide in AN (Strumila et al. 2022), and data narrative review suggested that zinc deficiency may contribute to the pathogenesis of AN via glutaminergic and GABAergic signaling (Mitchell et al. 2023). Another review highlighted the potential important role of screening and micronutrient supplementation in patients with AN (Jowik et al. 2021).

Data on micronutrient intake and deficiencies specifically in pediatric AN remain limited. Few studies evaluated the status of micronutrients (vitamins, trace elements, electrolytes) in children with AN and most included small cohorts, with incomplete laboratory assessments and sparse data (Setnick 2010; Modan‐Moses et al. 2015). Therefore, extensive screening for micronutrient deficiencies should be part of the phenotyping of pediatric patients with AN before the impact of supplementation is explored. The first aim of the present study was to provide a comprehensive description of micronutrient status in a highly phenotyped pediatric population with AN. The second aim was to investigate group differences based on the subtype of AN on laboratory parameters, in particular on micronutrient status. Since some published data suggest that AN‐R and AN‐BP could differ by their eating patterns (Elran‐Barak et al. 2014), they may consequently differ in their micronutrient profile (Hanachi et al. 2019).

## 2 Materials and Methods

### 2.1 Study Design and Population

This single‐center, observational and retrospective study was conducted at a university hospital's eating disorder reference center. All outpatients < 18 years of age who were diagnosed with AN according to DSM‐5 criteria between February 2016 and June 2022 were eligible (American Psychiatric Association et al. 2013). Patients with missing micronutrient data were excluded (incomplete data); also excluded were patients who declared the use of supplements.

According to national guidelines, patients underwent a standardized assessment during which the patient's medical history and anthropometric data were recorded, a psychiatric assessment was made, blood samples were taken, and bone health was assessed (Haute Autorité de Santé 2026). The results of this standardized assessment are collected and entered into the reference center's computerized database. According to French law, all patients gave their consent during the initial consultation for their anonymized data to be used from this database. The database complies with the French national data protection authority (*Commission nationale de l'informatique et des libertés*, CNIL) methodology (MR‐004) and is registered (number 19‐041). The protocol was approved by the local ethics committee.

### 2.2 Clinical Parameters

Anthropometric including age and sex assigned at birth were collected from the database. During the first consultation, the same practitioner measured the patients' height and weight under standardized conditions: fasting weight was measured in the morning on the same calibrated scale, and height was measured using a height chart. Weight was expressed as an absolute value (kg) and a percentile, and height as an absolute value (cm) and a Z‐score, according to the national references. Body Mass Index (BMI) was expressed as an absolute value, as well as according to the International Obesity Task Force (IOTF) reference curves, as recommended in the French national growth charts, allowing comparison with adult BMI (Courbes AFPA‐CRESS/Inserm‐CompuGroup Medical 2018). Weight kinetics were expressed as a percentage and determined using clinical data collected during the initial interview, based on weight prior to the onset of AN and the lowest weight recorded in the patient's medical history, provided by the referring physician or reported by the patient. All patients had their heart rate (HR), as well as their systolic (SBP) and diastolic (DBP) blood pressure and temperature (T°), measured after 15 min of rest. HR, SBP, and DBP were interpreted according to international standards for children and adolescents aged 6–17 years (Xi et al. 2016).

### 2.3 Laboratory Parameters

All blood samples were collected after an overnight fast. All analyses were performed in the laboratories of our university hospital. Routine biochemistry analyses were only performed on the biochemistry platform of the university biochemistry laboratory.

Selenium levels were determined using Inductively Coupled Plasma–Mass Spectrometry (ICP–MS) on an Elan DRCe instrument (PerkinElmer, Waltham, MA, USA) in Dynamic Reaction Cell (DRC) mode, with oxygen as the reaction gas and ^78^Se as the chosen isotope. The internal standard was rhodium. Inductively coupled plasma optical emission spectrometry (ICP‐OES; 5110 instrument, Agilent, Santa Clara, CA, USA) was used to determine plasma copper and zinc levels. The emission wavelengths were 213.857 and 327.395 nm, respectively.

Vitamin A and vitamin E levels were measured using high‐performance liquid chromatography coupled with spectrophotometric detection on an H‐Class Waters chromatography system (Waters, St Quentin‐en‐Yvelines, France). Vitamins B9 and B12 levels were measured using an electrochemiluminescence immunoassay (cobas pro, Roche, France). Vitamin D levels were measured using a chemiluminescence immunoassay (LIAISON 25 OH Vitamin D TOTAL Assay; DIASORIN, Antony, France).

### 2.4 Body Composition

As body composition may differ between AN‐R and AN‐B, we decided to include dual‐energy X‐ray absorptiometry (DEXA) measurements as a covariate in the analysis of deficiencies, by collecting values of fat mass (FM) and fat‐free mass (FFM). DEXA was performed using a Hologic Discovery W “fan beam” system (software version 12.6.1:7; Hologic Inc., Newark, DE 19702, USA). Mineral bone health was evaluated according to International Society for Clinical Densitometry (ISCD) guidelines. As recommended for children, two sites were evaluated: the lumbar spine and the whole body excluding the head. Whole‐body bone mineral content (BMC) was assessed (Crabtree et al. 2014). The results were expressed as absolute values (as well as Z‐scores for BMC), using device reference curves (Braillon et al. 2002). Low BMC was defined as a Z‐score lower than −2 standard deviations (SD).

### 2.5 Statistical Analysis

All statistical analyses were performed using SPSS software, version 20 (SPSS Inc., Chicago, Il, USA). Continuous variables were expressed as means ± SD. Demographic characteristics and biological concentrations were compared between the AN‐R and AN‐B groups using Welch's *t*‐test for independent samples. A Benjamini–Hochberg correction was applied to account for multiple comparisons. Hedge's g method was used to estimate the effect size. A multiple linear regression was done to determine a potential correlation between micronutrients' plasmatic levels and the body composition. The BMI, %FM, and BMC were included in the analysis as covariables. A *p*‐value of less than 0.05 was used as the significance threshold.

## 3 Results

### 3.1 Clinical and General Laboratory Parameters

**FIGURE 1:**
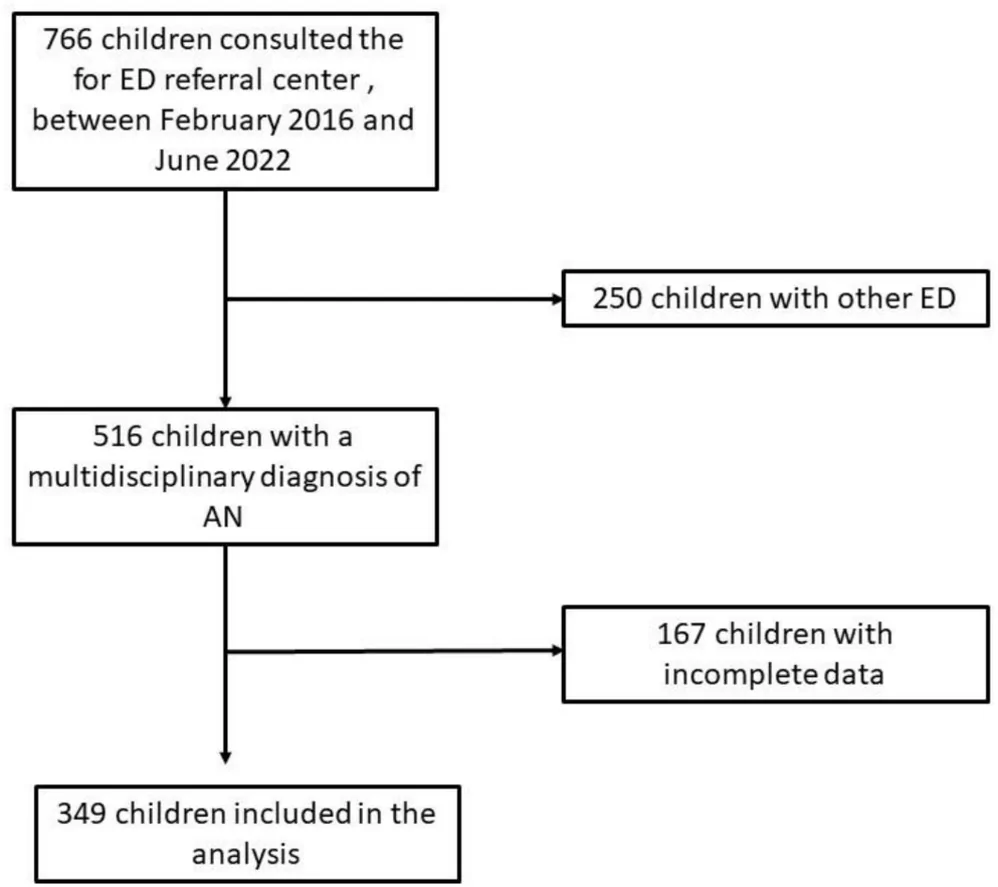
Study flowchart AN, anorexia nervosa; ED, Eating Disorder.

Between February 2016 and June 2022, 766 patients who consulted at the reference center for eating disorders, of whom 516 were diagnosed with AN. Among these, 349 (67.6%) underwent a complete assessment including micronutrient assessment; none reported taking micronutrient supplements, and therefore all were included (Figure 1). Their age ranged from 7 to 17 years, and the mean ± SD age was 14.7 ± 1.8 years (Table 1). The mean ± SD duration of illness at the time of the micronutrient assessment was 13.3 ± 9.8 months, and the sex ratio was 1:13. A total of 319 (91.4%) patients had AN‐R, who were significantly younger than those who had AN‐BP with a moderate size effect (14.7 ± 1.8 vs. 15.5 ± 1.4 years, *p* < 0.05, *g* = 0.451; Supplement 1 in the Supporting Information).

**TABLE 1:** Characteristic of overall population.

Name (unit)	*N*	Mean (SD)	Deficiencies, %
Weight (kg)	349	41.1 (7.4)	
Weight (percentile)	349	14.7 (20.3)	70.2
Height (cm)	349	160.5 (8.3)	
Height (Z‐score)	349	−0.1 (1.3)	13.2
BMI (Kg/m^2^)	349	15.9 (2.1)	
IOTF	349	16.7 (1.1)	76.5
Weight before AN (kg)	135	50.9 (8.7)	
Height before AN (cm)	136	159.3 (6.7)	
BMI before AN (kg/m^2^)	134	20.0 (2.8)	
Weight loss (kg)	141	9.3 (6.5)	
Weight loss (%)	141	18.2 (10.7)	
Weight loss kinetics (kg/month)	133	3.0 (2.0)	
Weight loss kinetics (%/month)	133	5.6 (3.2)	
FFM (kg)	210	29.9 (4.6)	
FM (kg)	210	8.1 (3.5)	
FM (%)	210		
Total BMC (kg)	190	1.7 (318)	
Total BMC (Z‐score)	190	0.2 (1.3)	5.3
Spine BMC (g)	190	44.0 (11.0)	
Spine BMC (Z‐score)	124	−0.8 (1.5)	17.7

Abbreviations: AN, anorexia nervosa; BMC, Bone Mineral Content; BMI, Body Mass Index; FFM, Fat Free Mass; FM, Fat Mass; IOTF, International Obesity Task Force; SD, Standard Deviation.

**FIGURE 2:**
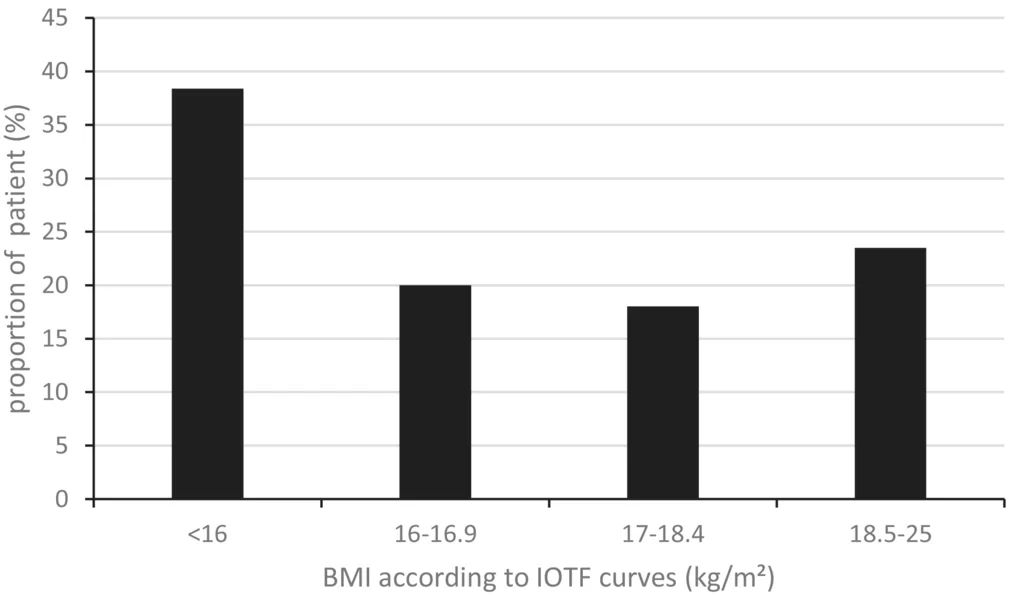
Distribution according to Body Mass Index (BMI); International Obesity Task Force (IOTF).

At the initial consultation at the reference center, the mean ± SD BMI was 15.9 ± 2.1 kg/m^2^, corresponding to 16.7 kg/m^2^ on the IOTF curve; 267 patients (76.5%) were diagnosed as being malnourished, including 204/267 (76.4%) as severely malnourished (Figure 2). The mean ± SD absolute BMI value was significantly lower in the AN‐R with a moderate size effect (15.8 ± 1.9 kg/m^2^ vs. 16.9 ± 3.3 kg/m^2^, *p* < 0.05, *g* = 0.534), but did not differ significantly from that in the AN‐BP group according to the IOTF reference curves (Supplement 1 in the Supporting Information). The mean ± SD weight loss at the time of micronutrient assessment was 18.2% ± 10.7% (Table 1), with no significant difference between the two subgroups (Supplement 1 in the Supporting Information).

Laboratory assessment of nutritional status found normal albumin and low free triiodothyronine (FT3) levels in 173 patients (54.6%), and low Insulin‐like Growth Factor‐1 (IGF‐1) levels in 284 patients (81.4%). All routine laboratory assessments are presented in Supplement 2 in the Supporting Information.

### 3.2 Micronutrients and Vitamins

According to the trace element assessment, deficiencies in magnesium, selenium, copper, and zinc were found in 205 (67%), 82 (23.5%), 56 (18.4%), and 10 (3.4%) patients, respectively (Table 2).

**TABLE 2:** Micronutrients profile of the population.

Name (unit)	*N*	Reference values	Mean (SD)	Deficiencies, % (*N*)	High values, % (*N*)
Vitamin A (μmol/L)	206	0.9–2.9	1.65 (0.42)	29.1 (60)	1.5 (4)
Vitamin D (nmol/L)	340	50–125	79 (28)	10.3 (35)	4.4 (15)
Vitamin E (μmol/L)	199	16–30	24.9 (5.1)	1.5 (3)	13.1 (26)
Vitamin B12 (nmol/L)	346	189–883	471 (225)	2.9 (10)	5.2 (18)
Vitamin B1 (nmol/L)	332	66–200	123 (25.2)	1.8 (6)	0.6 (2)
Vitamin B9 (μg/L)	345	2.3–13.7	7.1 (3.3)	1.7 (6)	41 (14)
Vitamin B6 (nmol/L)	95	15–73	83 (45)	0.0 (0)	51.6 (49)
Selenium (μg/L)	349	70–120	83 (15)	23.5 (82)	0.9 (4)
Copper (mg/L)	304	0.88–1.20	0.78 (0.20)	18.4 (56)	4.3 (13)
Zinc (mg/L)	348	0.62–0.99	0.74 (0.16)	3.4 (12)	24.1 (84)
Ferritin (μg/L)	343	20–250	90 (70)	11.7 (40)	6.1 (21)
Phosphorus (mmol/L)	349	1.13–2.00	1.26 (0.15)	18.9 (66)	0.0 (0)
Calcium (mmol/L)	346	2.10–2.55	2.41 (0.10)	0.0 (0)	3.5 (12)
Magnesium (mmol/L)	306	0.86–1.17	0.84 (0.06)	67.0 (205)	0.0 (0)
Potassium (mmol/L)	348	3.4–4.7	4.0 (0.4)	5.2 (18)	4.6 (16)
Sodium (mmol/L)	349	135–145	140 (2)	0.3 (1)	0.0 (0)

Abbreviation: SD, Standard Deviation.

For fat‐soluble vitamins, deficiencies in vitamin A, D, and E were found in 60 (29.1%), 35 (10.3%) and 3 (1.5%) patients, respectively; for hydro‐soluble vitamins, deficiencies in vitamin B12, B1, B9, and B6 were found in 10 (2.9%), 6 (1.8%), 6 (1.7%), and 0 patients, respectively. Forty (11.7%) patients had a low ferritin level (Table 2 and Supplement 3 in the Supporting Information).

Within the cohort, 10.6% of patients presented no deficiencies, 89.4% presented at least one deficiency; 28.9% had two deficiencies and 22.3% had three deficiencies (Supplement 4 in the Supporting Information).

AN‐R/AN‐BP subgroup analysis found no significant difference in these parameters between the two groups, except for potassium and calcium, which were significantly lower in AN‐BP. In addition, there was a trend toward a lower phosphorus level in AN‐BP (Table 3).

**TABLE 3:** Comparison of the AN‐R and AN‐BP subgroups.

Name (unit)	AN‐R		AN‐B		Raw *p*	Adjusted *p* (BH)	Hedges' *g*
	*N*	Mean (SD)	*N*	Mean (SD)			
Ferritin (μg/L)	314	92 (71)	28	65 (65)	0.057	0.171	0.38
Selenium (μg/L)	319	82.3 (15.4)	30	82.4 (16)	0.857	0.898	−0.01
Copper (mg/L)	277	0.8 (0.2)	27	0.8 (0.2)	0.653	0.812	0.00
Zinc (mg/L)	318	0.7 (0.2)	30	0.7 (0.2)	0.687	0.812	0.00
Vitamin B1 (nmol/L)	302	122.4 (26)	30	124 (21.6)	0.693	0.812	−0.006
Vitamin B6 (nmol/L)	85	84.7 (47.1)	10	70.3 (24.5)	0.137	0.343	0.31
Vitamin B12 (nmol/L)	316	471 (223)	30	470 (254)	0.898	0.898	0.00
Vitamin B9 (nmol/L)	315	7.1 (3.3)	30	7 (2.7)	0.878	0.898	0.03
Vitamin D (nmol/L)	312	79 (28)	28	74 (24)	0.322	0.603	0.18
Vitamin A (μmol/L)	191	1.65 (0.41)	15	1.76 (0.57)	0.468	0.704	−0.26
Vitamin E (μmol/L)	184	25 (5.1)	15	24 (5.6)	0.493	0.704	0.10
Potassium (mmol/L)	**318**	**4 (0.4)**	**30**	**3.8 (0.3)**	**0.002**	**0.015**	0.51
Calcium (mmol/L)	**316**	**2.42 (0.1)**	**30**	**2.36 (0.08)**	**0.001**	**0.015**	0.61
Phosphorus (mmol/L)	319	1.27 (0.15)	30	1.21 (0.14)	0.052	0.171	0.40
Magnesium (mmol/L)	280	0.84 (0.06)	26	0.85 (0.05)	0.312	0.603	−0.17

Abbreviations: AN‐BP, Anorexia with Binge Eating and Purging; AN‐R, Anorexia Nervosa with Restrictive Eating; BH, Benjamini–Hochberg; SD, Standard Deviation. Bold values indicate statistically significant differences between groups after adjustment for sample size (*p* < 0.05).

### 3.3 Body Composition

According to the DEXA results, 10 patients (5.3%) had low bone mass according to the BMC Z‐score, and 22 patients (17.7%) had low bone mass according to the lumbar spine BMC Z‐score. According to the AN subgroup, there was no significant difference for either BMC, FM, and FFM (Supplement 1 in the Supporting Information). There was no significative correlation between micronutrient deficiencies and BMI, BMC, or %FM.

## 4 Discussion

The present study found that micronutrient deficiencies were very common in the pediatric AN population, despite the shorter duration of illness compared to adults (2–16 years in adult series) (Achamrah et al. 2017; Hanachi et al. 2019). Children with AN had a deficiency profile characterized by a predominance of selenium and copper among trace elements, and vitamin A and D among liposoluble vitamins. The identification of these deficiencies highlights how dietary restrictions and choices affect patients beyond growth. Furthermore, patients with AN‐R did not differ from those with AN‐BP in terms of overall nutritional status or micronutrient status.

The present study confirms a high prevalence of undernutrition and severe undernutrition in children with AN (Hankard et al. 2012), as defined by national guidelines (Alexandre 2019). This finding is consistent with the frequent prevalence of laboratory markers of malnutrition, such as plasmatic IGF‐1 and leptin. More generally, owing to the lack of data in children and as nutritional environments and habits vary by country, we focused on two of the largest studies conducted in France among adults to compare the present study (Achamrah et al. 2017; Hanachi et al. 2019). These two studies of adult patients produced conflicting results regarding micronutrient status; while Achamrah et al. found a high prevalence of selenium deficiency without copper deficiency (Achamrah et al. 2017), Hanachi et al. found a high prevalence of deficiencies in all trace elements, particularly zinc (Hanachi et al. 2019). The two studies differed in terms of sample size (153 and 374 patients, respectively), but primarily in terms of the nutritional status of their patients, with a higher mean BMI in the Achamrah et al. cohort (17 ± 2.6 kg/m^2^ vs. 12.5 ± 1.7 kg/m^2^). Although the mean BMI of the patients included herein (expressed according to the IOTF curves) was close to those of the patients included in the study reported by Achamrah et al. (2017); Hanachi et al. (2019). It could be hypothesized that the prevalence of micronutrient deficiency reflects the severity of low‐calorie food pattern rather than the severity of malnutrition, since BMI did not correlate with specific micronutrient levels herein (data not shown).

There are currently no recommendations regarding micronutrient screening in AN, neither in adult or the pediatric population, and published data on the functional impact of micronutrient deficiency in AN are scarce, and only concern selenium. In the remainder of this discussion, we present arguments regarding the serious consequences of micronutrient deficiencies in AN and other models. Deficiency in plasmatic selenium was the most prevalent trace element deficiency herein and is now well known to play a role in the antioxidant response, immune system function, thyroid hormone synthesis, and brain function (Avery and Hoffmann 2018; Drutel et al. 2013; Guillin et al. 2019; Rayman 2000). Selenium acts through an important selenoprotein: glutathione peroxidase. Malnutrition is accompanied by increased oxidative stress, which is a maintaining factor in the course of AN (Agnello et al. 2012). Furthermore, plasma selenium levels appear to influence psychiatric comorbidities in malnourished patients, as patients with low dietary selenium intake exhibit more anxiety or depressive symptoms and benefit from selenium supplementation (Benton and Cook 1991; Hawkes and Hornbostel 1996). In addition, a recent study of 153 patients with AN found a positive correlation between selenium deficiency, earlier onset of AN, disruption to reward processing, and an increased risk of suicide (Strumila et al. 2022). While the relationship between selenium deficiency, oxidative stress, and the psychic phenotype in AN requires further clarification, selenium appears to be a significant factor in the pathophysiology of AN. Conversely, chronic selenium deficiency in AN can lead to significant cardiac complications. Keshan disease, a model of chronic selenium deficiency, was first described in a region with low selenium levels in the soil and local foodstuffs. Histological findings implied myocardial pallor presenting as extensive patchy fibrosis, necrosis, and myocytolysis (Loscalzo 2014). This “white muscle disease” was subsequently described in a case report of AN with severe undernutrition, suggesting the potential benefits of selenium supplementation in children with AN (Ishihara et al. 1999). Finally, although selenium status primarily depends on dietary intake, other studies have suggested that genetic polymorphisms could impact plasmatic selenium levels and its response to supplementation (Mao et al. 2016; Combs 2015). Combined with previous data, exploring the impact of genetic premorbid selenium levels is important.

For the other deficiencies found herein, the results have to be interpreted using data from other models. For instance, copper deficiency was the second most common micronutrient deficiency observed, but interestingly in AN this may not only be due to insufficient intake. A high‐fiber vegan diet could act as a copper trap, decreasing its absorption rate in enterocytes (Gibson 1994). The high prevalence of vegetarian diets among adolescents with AN (Sergentanis et al. 2020), due to low‐calorie food pattern and/or environmental beliefs could explain the high prevalence of copper deficiency in the present study. Like selenium, copper is an important catalytic cofactor for several redox reactions involved in different pathways, such as iron metabolism, neurotransmission, bone metabolism, and immune signaling (Gibson 1994; Prohaska 2014). Despite the lack of data for children with AN, the severe consequences of copper deficiency described in other diseases, such as hereditary copper deficiency (Menkes disease) or prolonged dependence on parenteral nutrition (Tümer and Møller 2010; Namjoshi et al. 2018) highlight the importance of exploring the impact of copper deficiency in AN. Children with Menkes disease exhibit progressive brain atrophy, demyelination lesions and intracranial vessel abnormalities early in life. Later in life, myelopathy and peripheral neuropathy are found in adults with copper deficiency (Taylor et al. 2017). Furthermore, copper deficiency can lead to anemia, cytopenia and skeletal abnormalities, particularly in pediatric cohorts receiving artificial nutrition without copper supplementation (Leite et al. 2021; Jacobson et al. 2017; Shike 2009; Kodama et al. 2011).

In this study, zinc deficiency has not been found at high levels compared to adults (Hanachi et al. 2019). However, there is evidence in favor of zinc supplementation, since zinc deficiency impacts both food intake and eating behavior. In malnourished children, for example, plasma zinc levels are a determinant of growth and appetite, and reaching and maintaining normal plasma zinc levels leads to improved appetite and nutritional status (Chao et al. 2018). Furthermore, a recent meta‐analysis found that zinc supplementation improves height prognosis in young children with diarrhea (Mayo‐Wilson et al. 2014). In AN, zinc supplementation could promote weight gain, as demonstrated by a small interventional study of malnourished adult patients (Birmingham et al. 1994). The authors suggest that normal levels of zinc improve the GABA neurotransmission system, which is impaired in AN and involved in the so‐called “inhibitory” regulation of brain activity (Birmingham and Gritzner 2006).

Given the previously described alteration to the structure and strength of bones, patients with AN can be considered a population at risk of bone fragility and fractures (Clarke et al. 2021). Deficiency in vitamin D is an important risk factor for altered bone metabolism, particularly during adolescence, which is a critical period for bone accrual. Surprisingly, insufficient vitamin D levels (defined as plasma values below 50 nmol/L) were found in only 10.3%. This is much lower than that observed in a recent European cohort study of the general population in which the prevalence of vitamin D deficiency among 3528 adolescents (1845 girls) was approximately 15% (Moreno et al. 2014). Furthermore, the results presented herein do not align with those of a study reported by Hanachi et al. who included adults with AN and who found that 54% of participants had vitamin D deficiency (Hanachi et al. 2019). However, according to DEXA, total BMC and spine BMC lower than −2SD were found in 5% and 18% of children with AN. This suggests that vitamin D deficiency is only one factor contributing to osteopenia in children with AN. Interestingly, a review found no relationship between vitamin D and bone content in adolescents with AN, probably because vitamin D plasma levels were higher than in controls and other factors are involved in osteopenia in AN, such as low IGF‐1 and low estrogen levels (Misra and Klibanski 2011). This result could be explained by higher dairy product intake in children with AN and/or systematic vitamin D supplementation in the primary care of adolescents. According to recommendations from the French National Authority for Health (*Haute Autorité de Santé*, HAS), vitamin D supplementation should be initiated when levels are below 75 nmol/L (Bacchetta et al. 2022); herein, approximately half of the patients had levels below this threshold, which may have led to unreported supplementation.

The present study does have some limitations. Data on micronutrients were missing for 167 (32%) patients, which could be interpreted as being quite important. However, there are several potential reasons for this, including referral of the patient to pediatric services for severe malnutrition, difficulties in blood sampling, and problems in the analysis of the sample. The prevalence of deficiencies may have been underestimated due to memory bias regarding supplementation. Furthermore, the main limitation is that we only provide laboratory data without evaluating its clinical consequences. Nevertheless, to our knowledge, this is the largest pediatric cohort focusing on the status of plasma micronutrients and evaluating the impact of AN subtypes (AN‐R and ‐BP). Furthermore, to ensure the most accurate interpretation, our laboratory's normal values for children and adolescents were aligned with those of other European pediatric cohorts (Ghosh et al. 2022; Al Fify et al. 2022; Li et al. 2021).

## 5 Conclusion

Despite a shorter disease duration compared to published adult cohorts, selenium, copper, and vitamin A deficiencies are the most frequent, with no difference between AN‐R and AN‐BP subtypes. However, more research is needed on the micronutrient status of children with AN, especially regarding the association between laboratory data and clinical consequences. Interventional studies on specific supplementation need to be conducted to determine the potential benefits of systematic micronutrient supplementation.

## Author Contributions

**Diane Morfin:** data curation, writing – review and editing. **Zenaida Iordan:** writing – original draft, writing – review and editing, conceptualization, data curation, methodology, investigation, formal analysis, visualization. **Marion Bernetiere:** data curation, formal analysis, writing – review and editing. **Marie Fauchet:** data curation, formal analysis, writing – review and editing. **Pierre Poinsot:** data curation, conceptualization, methodology, investigation, formal analysis, visualization, supervision, writing – original draft, writing – review and editing. **Bérénice Segrestin:** data curation, writing – review and editing, conceptualization. **Noël Peretti:** writing – review and editing, conceptualization.

## Lived Experience Involvement Statement

No specific efforts were undertaken to involve persons with lived experience in the study design or execution, or in the preparation of this manuscript.

## Funding

The authors have nothing to report.

## Conflicts of Interest

The authors declare no conflicts of interest.

## Supporting information


**Supplement 1.** Characteristics of the AN‐R and AN‐BP subgroups.
**Supplement 2**. Boxplot of status in micronutrients.
**Supplement 3**. Biological characteristics of the studied population—General parameters.
**Supplement 4**. Reference values for biological data.
**Supplement 5**. Percentage of patients regarding cumulative deficiencies.

## Data Availability

The data that support the findings of this study are available on request from the corresponding author. The data are not publicly available due to privacy or ethical restrictions.
