# Supplementary material for: Micronutrient Status in Children Suffering From Anorexia Nervosa: A Cohort of 349 Patients in a Referral Center for Eating Disorders
Source: Int J Eat Disord. 2026 May 17;59(9):2004–12. doi: 10.1002/eat.70120 (PMC13536237; doi:10.1002/eat.70120)
Supplement: Supplementary file 1 [file EAT-59-2004-s001.docx]

# Supplementary data:

| **Supplement 1. Characteristics of the AN-R and AN-BP subgroups** | | | | | |
| --- | --- | --- | --- | --- | --- |
|  |  | AN-R  (n=319) | AN-BP  (n=50) | t-Test (p*) | |
| Age | (Years) | **14.7 ± 1.8** | **15.5 ± 1.4** | | **<0.05** |
| Weight | (kg) | **40.8 ± 7.2** | **43.6 ± 8.9** | | **<0.05** |
| Height | (cm) | 160.5 ± 8 | 160.7 ± 8 | | NS |
| BMI | (kg/m²) | **15.77 ± 1.95** | **16.89 ± 3.26** | | **<0.05** |
| IOTF |  | 16.7 ± 1.1 | 17 ± 1.2 | | NS |
| Weight loss | (Kg) | 9.2 ± 6.5 | 10.1 ± 7.6 | | NS |
| Weight loss | (%) | 18.2 ± 10.6 | 18.8 ± 11.9 | | NS |
| SBP | (mmHg) | 105 ± 13 | 119 ± 15 | | NS |
| DBP | (mmHg) | 64 ± 7 | 65 ± 8 | | NS |
| HR | (bpm) | **61 ± 15** | **68 ± 16** | | **<0.05** |
| Temperature | (°C) | **36.2 ± 0.6** | **36.5 ± 0.6** | | **<0.05** |
| FFM DXA | (Kg) | 29.9 ± 4.6 | 29.9 ± 4.4 | | NS |
| FM DXA | (Kg) | 8.1 ± 3.4 | 8.1 ± 4.4 | | NS |
| BMC total | (G) | 1679 ± 317 | 1689 ± 335 | | NS |
| Measured REE | (kCal/d) | 1070 ± 258 | 1079 ± 347 | | NS |
| RQ |  | 0.85 ± 0.1 | 0.84 ± 0.09 | | NS |
| AN-R, Anorexia Nervosa with Restrictive Eating; AN-BP, Anorexia with Binge Eating and Purging; bpm, beats per minute; BMC, Bone Mineral Content; BMI, Body Mass Index; C, Celsius; DBP, Diastolic Blood Pressure; DXA, Dual-energy X-ray Absorptiometry; FFM, Free Fat Mass; FM, Fat Mass; HR, Heart Rate; IOTF, International Obesity Task Force; REE, Resting Energy Expenditure; RQ, Respiratory Quotient; SBP, Systolic Blood Pressure  *p<0.05, significant difference according to T-test  Bold values indicate statistically significant difference between the two subgroups | | | | | |

**Supplement 2. Boxplot of status in micronutrients**

|  |  |  |  |
| --- | --- | --- | --- |
|  |  |  |  |
|  |  |  |  |
|  |  |  |  |

The box represents the interquartile range (IQR), extending from the first quartile to the third quartile. The black horizontal indicates the median blood values tests of the cohort. The red horizontal indicates the lower limit of the normal blood tests values. The whiskers extend to the minimum values that are not considered outliers. Circles (°) represent mild outliers, defined as values located between 1.5 and 3 times the IQR above or below the box. Values are represented individually by boxplots in mg/l (Copper, Zinc), μg/L (Ferritin, Selenium and Vitamin B9), µmol/L (Vitamins A and E), mmol/L (Calcium, Magnesium, Phosphorus, Potassium and Sodium), nmol/L (Vitamin B1, B6, D) and ng/L (Vitamin B12)

| **Supplement 3. Biological characteristics of the studied population – General parameters** | | | | | | |
| --- | --- | --- | --- | --- | --- | --- |
| **Name (Unit)** | **N** | **Normal values** | **Average (SD)** | **Min-Max** | **% of low values (N)** | **% of high values (N)** |
| Albumin (g/L) | 338 | 35-50 | 47.5 (3.8) | 34-59 | 0.3 (1) | 22.7 (70) |
| Prealbumin (g/L) | 336 | 0.16-0.38 | 0.24 (0.05) | 0.11-0.38 | 4.8 (18) | 0 (0) |
| IGF-1 (µg/L) | 340 | N/A | 191 (97) | 26-577 | 81.4 (284) | 0 (0) |
| Glucose (mmol/L) | 347 | N/A | 4.2 (0.7) | 2.5-12.8 | 29.4 (102) | 1.7 (6) |
| Urea (mmol/L) | 346 | 2,6-6,8 | 4.7 (1,5) | 1.3-13.4 | 6.1 (21) | 7.2 (25) |
| Creatinine (µmol/L) | 346 | 40-90 | 64 (12) | 28-106 | 1.2 (4) | 2 (8) |
| GFR Schwartz (ml/min) | 346 | N/A | 138 (27) | 81-286 | 5.5 (19) | 0 (0) |
| ASAT (UI/L) | 341 | <33 | 29 (13) | 13-105 | 0 (0) | 15.2 (52) |
| ALAT (UI/L) | 340 | 8-24 | 27 (14) | 6-237 | 0 (0) | 38.5 (131) |
| Triglycerides (mg/dL) | 337 | 35-150 | 68 (24) | 26-187 | 3 (10) | 0.3 (1) |
| Cholesterol (mg/dL) | 337 | 131-201 | 176 (37) | 101-428 | 7.4 (25) | 22.6 (76) |
| LDL-Cholesterol (mg/dL) | 327 | 65-120 | 105 (34) | 40-339 | 11.3 (37) | 23.2 (76) |
| HDL-Cholesterol (mg/dL) | 327 | >42 | 58 (12) | 31-110 | 10.4 (34) | 0 (0) |
| FT3 (pmol/L) | 317 | 3.3-7.6 | 3.5 (1.1) | 1.4-7.9 | 54.6 (173) | 0.3 (1) |
| Leptin (ng/L) | 337 | 5.42-20.62 | 3.08 (3.89) | <1-34 | 83.7 (282) | 0.6 (3) |
| Haemoglobin (g/L) | 340 | 120-160 | 130 (11) | 100-210 | 16.8 (57) | 0 (0) |
| Leucocytes (Giga/L) | 340 | 4-10 | 4.9 (1.52) | 2.33-13.65 | 31.5 (107) | 0.6 (2) |
| Platelets (Giga/L) | 339 | 150-400 | 230 (53) | 125-414 | 3.5 (12) | 0,9 (3) |
| TP | 324 | 70-100 | 85 (9) | 31-100 | 5.6 (18) | 0 (0) |
| FSH (UI/L) | 336 | N/A | 3.8 (2.6) | 0.2-11.7 | 41.4 (139) | 0 (0) |
| LH (UI/L) | 336 | N/A | 2.07 (2.93) | 0.02-18.13 | 64.6 (217) | 0 (0) |
| Estradiol (pmol/L) | 313 | >50 | 86 (132) | 16-1102 | 54.3 (167) | 0 (0) |
| Testosterone (nmol/L) | 18 | >0.3 | 4.62 (7.54) | 0.12-30.72 | 44.4 (8) | 0 (0) |

%, Percentage; ALAT, alanine aminotransferase; ASAT, aspartate aminotransferase; FSH, follicle-stimulating hormone; FT3, Free Triiodothyronine; GFR, Glomerular Filtration Rate; HDL, High Density Lipoprotein; IGF-1, Insulin-like Growth Factor-1; LDL, Low Density Lipoprotein; LH, luteinizing hormone; N, Number; N/A, Not Applicable; SD, Standard Deviation; TP, Total Protein.

The plasmatic IGF-1 was assessed in the hormonology laboratory by chemiluminescence on an IDS ISYS automated, calibrated against the WHO standard 02/254. Serum reference values are evaluated according to Bidlingmaier *et al*., 2013, considering age, sex, and the median reference value. FSH, LH, estrogen, and testosterone were assessed in the hormonology laboratory by immune-analysis on ARCHITECT i2000 (Abbott™) calibrated on WHO 92/510 standard values considering low values as under the minimum values of follicular phases. Renal clearance (normal values: boys 98-150 ml/min, girls 95.5-122.5 ml/min), was determined according to the Schwartz formula adapted to children using plasma creatinine in μmol/L, the size, age, and sex of the child.

**Supplement 4. Reference values for biological data**

| Undernutrition parameters | | |
| --- | --- | --- |
| C Reactive Protein | <5 mg/L | |
| Albumin | 35-50 g/L | |
| Prealbumin | 0.16-0.38 g/L | |
| IGF-1 | Median | Extreme values |
| Male |  |  |
| 6-11 ans | 184.2 µg/L | 56.2 – 454.4 µg/L |
| 11-13 ans | 278.9 µg/L | 111.6 – 532.5 µg/L |
| 13-15 ans | 314.4 µg/L | 138.6 – 553.5 µg/L |
| 15-18 ans | 310.8 µg/L | 146.2 – 553.5 µg/L |
| Female |  |  |
| 6-11 ans | 165.8 µg/L | 42.0 – 452.6 µg/L |
| 11-13 ans | 262.7 µg/L | 92.6 – 533.4 µg/L |
| 13-15 ans | 296.2 µg/L | 115.9 – 554.2 µg/L |
| 15-18 ans | 291.3 µg/L | 120.5 – 554.2 µg/L |
| Blood ionogram |  | |
| Creatinine | 40-90 µmol/L | |
| GFR by Schwartz formula  Male  Female | 98-150 ml/min  95.5-122.5 ml/min | |
| Urea | 2.6-6.8 mmol/L | |
| Sodium | 135-145 mmol/L | |
| Potassium | 3.4-4.7 mmol/L | |
| Magnesium | 0.87-1.17 mmol/L | |
| Calcium | 2.1-2.55 mmol/L | |
| Phosphorus | 1.13-2 mmol/L | |
| Glucose  Male  Female | 3.3-5.5 mmol/L  3.9-5.9 mmol/L | |
| Hormonal blood tests |  | |
| TSH | 0.4-3.1 mUI/L | |
| FT3 | 3.4-5.2 pmol/L | |
| FT4 | 12-22 pmol/L | |
| Leptin |  | |
| Male | 2.45 – 6.55 ng/mL | |
| Female | 5.42 – 20.62 ng/mL | |
| Gonadotropic blood tests |  | |
| FSH  Male  Female | 1.1-7.2 UI/L  >3 UI/L | |
| LH  Male  Female | 1.3-7.8 UI/L  >1.8 UI/L | |
| Estradiol | >50 pmol/L | |
| Testosterone | >0.3 nmol/L | |
| Hepatic and blood clotting tests |  | |
| ASAT | <33 | |
| ALAT | 8-24 | |
| PT | >70% | |
| Lipid blood tests |  | |
| Triglycerides | 0.35-1.5 g/L | |
| Cholesterol | 1.31-2.01 g/L | |
| LDL-Cholesterol | 0.65-1.20 g/L | |
| HDL-Cholesterol | >0.42 g/L | |
| Nutriments |  | |
| Ferritin | 10-250 µg/L | |
| Zinc | 0.62-0.99 mg/l | |
| Copper | 0.88-1.20 mg/L | |
| Selenium | 70-120 µg/L | |
| Vitamin B1 | 66-200 nmol/L | |
| Vitamin B6 | 15-73 nmol/L | |
| Vitamin B9 | 2.3-13.7 µg/L | |
| Vitamin B12 | 189-883 nmol/L | |
| Vitamin D | 50-125 nmol/L | |
| Vitamin A | 0.9-2.9 µmol/L | |
| Vitamin E | 16-30 µmol/L | |

ALAT, alanine aminotransferase; ASAT, aspartate aminotransferase; FSH, Follicle-Stimulating Hormone; FT3, Free Triiodothyronine; FT4, Free Tetraiodothyronine; GFR, glomerular filtration rate ; HDL, High Density Lipoprotein; IGF-1, Insulin-like Growth Factor-1 ; LDL, Low Density Lipoprotein; LH, Luteinizing Hormone; PT, Prothrombin test; TSH, Thyroid Stimulating Hormone.

Supplement 5. Percentage of patients regarding cumulative deficiencies
